# Assessment of Silicone Rubber/Lead Oxide Composites Enriched with Bi_2_O_3_, WO_3_, BaO, and SnO_2_ Nanoparticles for Radiation Shielding Applications

**DOI:** 10.3390/polym15092160

**Published:** 2023-04-30

**Authors:** Mohammed Thamer Alresheedi, Mohamed Elsafi, Yosef T. Aladadi, Ahmad Fauzi Abas, Abdullrahman Bin Ganam, M. I. Sayyed, Mohd Adzir Mahdi

**Affiliations:** 1 Department of Electrical Engineering, King Saud University, P.O. Box 800, Riyadh 11421, Saudi Arabia; 2 Physics Department, Faculty of Science, Alexandria University, Alexandria 21511, Egypt; 3 Department of Physics, Faculty of Science, Isra University, Amman 11622, Jordan; 4 Department of Nuclear Medicine Research, Institute for Research and Medical Consultations (IRMC), Imam Abdulrahman bin Faisal University (IAU), P.O. Box 1982, Dammam 31441, Saudi Arabia; 5 Wireless and Photonics Research Centre, Universiti Putra Malaysia, Serdang 43400, Selangor, Malaysia

**Keywords:** heavy metal oxide nanoparticles, gamma ray shielding, thermal stability, radiation attenuation, half-value layer

## Abstract

This study aimed to prepare silicone rubber composites with heavy metal oxide nanoparticles for gamma ray shielding applications. Different heavy metal oxide nanoparticles were incorporated into the silicone rubber matrix, and the prepared composites were characterized for their thermal, mechanical, and radiation shielding properties. The density of the prepared SR samples ranged from 1.25 to 2.611 g·cm^−3^, with SR-2 having the highest density due to the presence of lead oxide. Additionally, the thermal stability of the materials improved with the addition of HMO nanoparticles, as indicated by TGA results. The prepared SR materials showed ultimate deformation displacement ranging from 14.17 to 21.23 mm, with the highest value recorded for SR-3 and the lowest for SR-2. We investigated the transmission factor (TF) of gamma rays through silicone rubber (SR) composites with different heavy metal oxide (HMO) nanoparticles. The addition of HMOs resulted in a decrease in TF values, indicating improved radiation shielding performance. The TF was found to be lowest in SR-5, which contained 15% of Bi_2_O_3_, WO_3_, BaO, and Zr_2_O_3_ each. The linear attenuation coefficient (LAC) of the SR samples was also evaluated, and it was found that the incorporation of HMOs increased the probability of photon interactions, leading to improved radiation protection effectiveness. The half-value layer (HVL) of the SR samples was also examined, and it was found that the addition of HMOs resulted in a significant reduction in HVL values, particularly at low energy levels. SR-5 had the lowest HVL among the group, while SR-2, SR-3, and SR-4 had higher HVL values. These results demonstrate the effectiveness of using HMOs in enhancing the radiation shielding properties of SR composites, particularly for low-energy gamma rays.

## 1. Introduction

In the past few years, as a result of significant advancements in technology, the number of clinical and commercial processes of ionizing radiation (including imaging techniques, radiotherapy, food and agricultural products, and many others) has expanded all over the world. However, even at modest levels, these kinds of radiation will inevitably have unfavorable impacts on living creatures, which will elevate the hazards to personal health. As a consequence of these potential unfavorable effects, the utilization of appropriate radiation shielding techniques plays an essential role in reducing health concerns and protecting people from the risks associated with radiation exposure [1,2,3,4,5,6].

Additionally, the demand for the exploration of some effective and dependable materials has been growing over the course of the past few years. Heavy metals, including lead, have been widely employed as a shield against ionizing radiation for a number of years now [7,8,9]. However, due to its limited chemical stability, low mechanical strength, potential toxicity, and heavy weight, the utilization of lead has been limited in a wide range of common applications. There is evidence that lead exposure can have a negative impact not only on human health but also on the surroundings in which it is located. However, academics are of the opinion that it is important to find suitable light-shielding substances as an alternative to the conventional use of lead shields. In this context, a variety of scientists have produced reinforced polymer composite materials that contain high atomic number components that have good radiation absorption capabilities [10,11,12,13].

These composites are easy to transport, inexpensive, flexible, lightweight, and non-toxic. Polymer composites have a wide range of applications across a variety of industries, as well as in the medical and scientific domains. In previous several years, a number of research facilities have put a significant amount of effort into developing polymers that include the necessary fillers in an effort to overcome the limitations that are associated with typical shielding materials [14,15].

Since it has great qualities, such as high flexibility, high thermal stability, weatherability, chemical resistance, bioactivity, optical clarity, etc., silicone rubber (SR) is considered one of the most significant artificial elastomers. SR is therefore used in modern technological fields such as aerospace, automotive, food processing, electrical insulators, medical instrument components, flexible electronics, and more [16,17,18].

Recent research has concentrated on utilizing nanoparticles (NPs) to improve some materials’ capability to act as shields. As opposed to microparticles, which are smaller and regarded as coarser, nanoparticles are regarded as fine particles. The smaller size of the NPs results in unique physical and chemical features, such as optical and radiation shielding qualities, within the matter they are implanted in. Nanoparticles can be utilized in the field of radiation shielding in addition to their existing uses in different computing and electronic products, transistors, magnetic data storage, superconducting, medical and healthcare applications, environmental remediation, and other technologies [19,20].

In this work, different silicone rubber samples with different HMO nanoparticles were prepared to investigate their thermal, mechanical, and radiation shielding properties at a wide range of energies.

## 2. Materials and Methods

### 2.1. Materials

#### 2.1.1. Silicone Rubber (SR)

Silicone rubber, in general, is a non-reactive, stable material that is resistant to harsh environments and retains its useful properties at temperatures ranging from −55 to 300 °C. Because of these properties and the ease and low cost of manufacturing, silicone rubber is found in a variety of applications, including radioprotective applications during radiological diagnosis [21]. The density of the present silicone rubber equals 1.181 g·cm^−3^ and has a viscosity of around 1,000,000 cps. Its mean components are silicone, carbon, oxygen, and hydrogen. The main supplier of silicone rubber is China. Silicone rubber can be processed by several systems, including platinum-catalyzed curing, peroxide-curing, or an oxime cure system [22].

#### 2.1.2. Heavy Metal Oxides Nanoparticles (HMOs-NPs)

**Figure 1:**
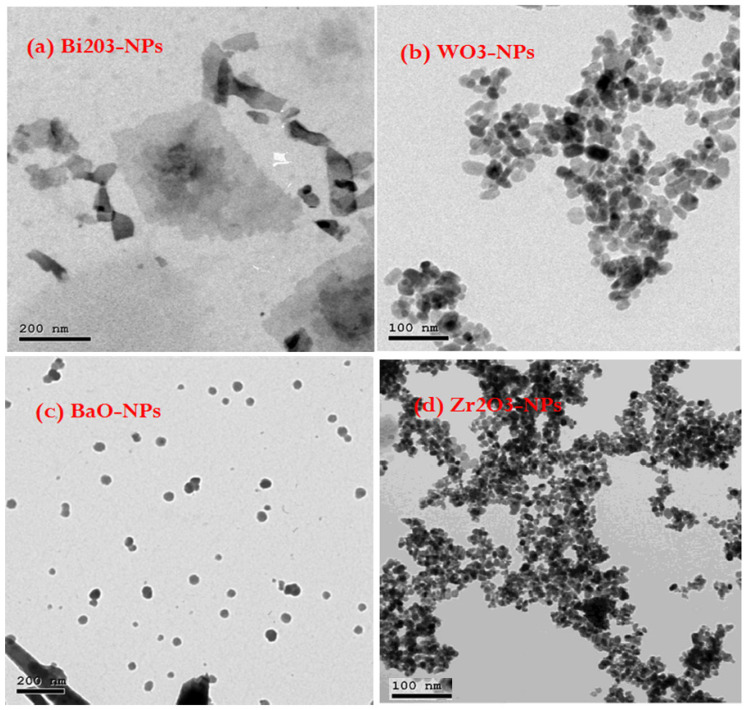
TEM images of present NPs, (**a**) Bi_2_O_3_-NPs, (**b**) WO_3_-NPs, (**c**) BaO-NPs, and (**d**) Zr_2_O_3_-NPs.

Bi_2_O_3_-NPs, WO_3_-NPs, BaO-NPs, and Zr_2_O_3_-NPs were chosen due to their higher density, as follows: 8.90, 7.16, 5.72, and 5.68 g·cm^−3^. As well, they have high absorption points (k-edges) at different energies, and NPs have higher homogenous distribution than the MPs inside the SR. These reasons improve the prepared SR samples against gamma ray radiations. TEM-images of presented NPs were performed using JEOL JEM-2100 high-resolution transmission electron microscope (JEOL, Ltd., Tokyo, Japan) at an accelerating voltage of 200 kV as shown in Figure 1 and the obtained properties of presented NPs were tabulated in Table 1. The average size of PbO-microparticles replaced with nanoparticles was 40 μm, its purity was 98.8%, it had 1.2% impurities and was purchased from EPPCO, in Al Arbaeen, Suez Governorate, Egypt.

### 2.2. SR Mixture Preparation

**Figure 2:**
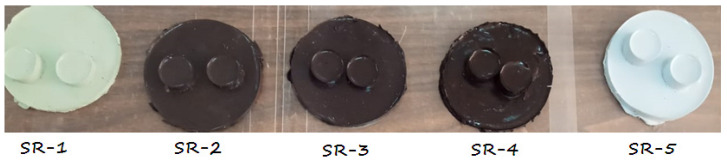
Different prepared SR samples.

The idea of preparing silicone rubber is the same as the previously studied preparation process [21,22], where silicone rubber is added in a container in the required quantity and the required oxides are added and stirred well for 10 min until the mixture becomes homogeneous, after which the silicone rubber hardener (a hardener of silicone rubber materials is a silane compound which comprises a 2-hydroxy-propionic acid alkyl ester radical) is added to the mixture at a rate of 5% of the amount of SR added. Then, the mixture is stirred for five min, and after that, the mixture is poured into several molds of different thicknesses and diameters and left to dry. The composition of the presently prepared silicone rubber mixtures is tabulated in Table 2, and Figure 2 shows the different prepared silicone rubber samples.

### 2.3. SR Mixture Characterization

**Figure 3:**
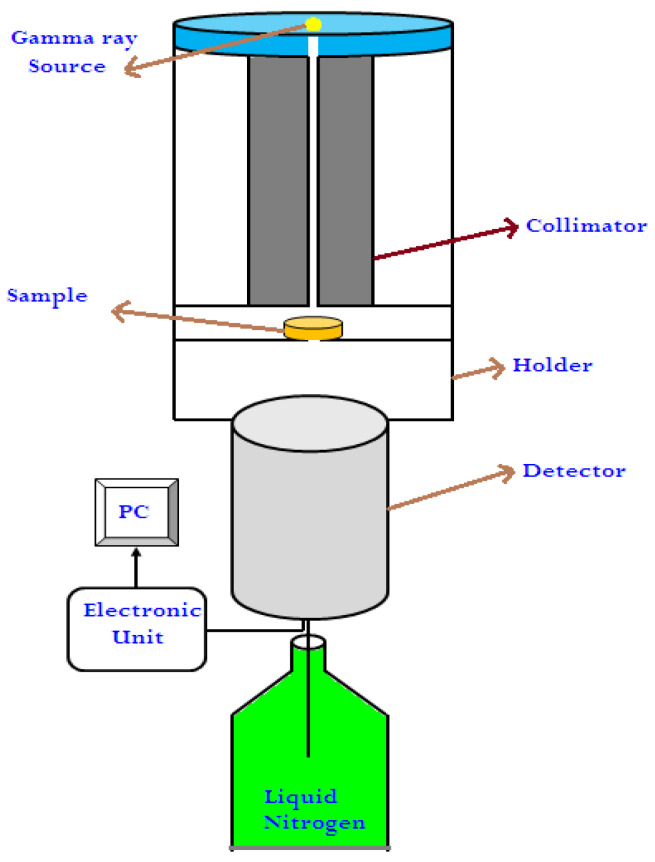
The experimental setup of the attenuation coefficient measurements.

The density of the present SR samples was measured experimentally using mass to volume of sample relation (ρ=M/V), as well as the thermal properties using TA machine (Thermal Analysis) [23] to determine the weight lost from raising the temperature from 30 to 900 °C by a rate of 20 °C per minute; this analysis is called thermogravimetric analysis (TGA). In addition, the stress–strain characteristic curve of the prepared SR mixtures was determined using Generic Compression stress vs. strain device [23], and the ultimate stress as well as deformation displacement were determined. Finally, the attenuation factor of the prepared SR mixtures was determined experimentally using HPGe detector [24] and different point sources [24], including Cs-137, Co-60, and Am-241. The mechanism of measuring is arranged in Figure 3, where the SR sample was placed between the detector and the source during the measurements to calculate the net count rate (N) due to the transmitted photons from the sample. Additionally, the measurement occurred in case without the SR sample, and the net count rate was calculated (N_0_). From these values, the linear-attenuation-coefficient (LAC) for sample of thickness (x) can be calculated by [25,26]:

(1) $$LAC=\frac{1}{x}\ln\frac{N_{0}}{N}$$

The other essential attenuator factors, such as HVL, TVL, and radiation shielding efficiency (RSE), which are discussed in [27,28,29,30,31,32,33], can be expressed by the following law:

(2) $$HVL=\frac{Ln(2)}{LAC}$$

(3) $$TVL=\frac{1}{LAC}$$

(4) $$RSE,\%=[1-\frac{I}{I_{0}}]\times 100$$

## 3. Results and Discussion

The density of advanced SR prepared samples was measured and evaluated according to Table 2, where the density was 1.25, 2.611, 2.555, 2.500, and 2.448 g·cm^−3^ for SR-1, SR-2, SR-3, SR-4, and SR-5, respectively. It is clear that the highest density was for the second sample (SR-2) because it contained lead oxide, which is the highest density of other added particle oxides, and the lower percentage of PbO in the silicone mixture correlates to a lower density, which is evident in the rest of the samples. Although the density of the samples plays an important role in the attenuation properties, in order to treat the environmental part of PbO and the side effect of adding it as a filler oxide, we compensated with corresponding oxides that have more than the k-edge. As well, the size of their particles is small in nano size because it has a higher chance of radiation attenuation.

**Figure 4:**
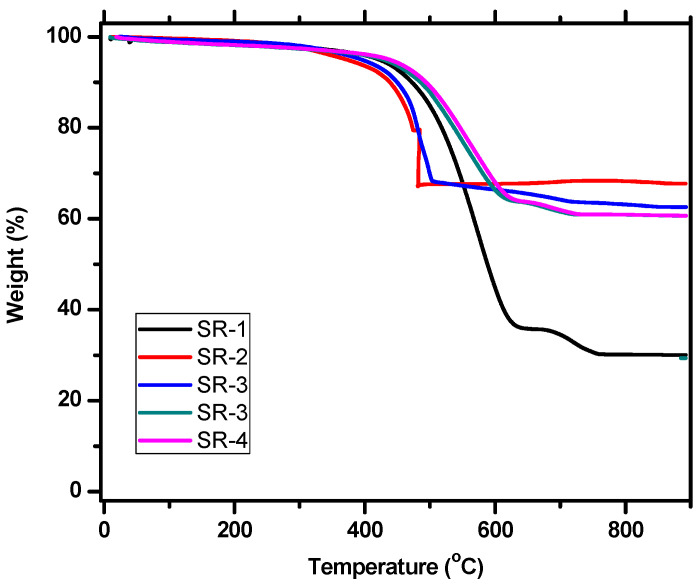
TGA of the SR samples in this work.

TGA was used for the prepared silicone rubber mixtures, where the weight loss with temperature increase was determined, as shown in Figure 4. The results showed that the addition of HMOs to silicone rubber improves the thermal properties of the compound: in the first sample that did not contain HMOs, its weight lost 75% when exposed to 900 °C, while the other four samples lost weight between 30 and 35% when exposed to the same temperature. From these results, it is clear that the addition of HMO nanoparticles improves the thermal stability of the materials studied in the current work, and this is a good addition to medical and industrial applications. Due to the different HMOs in the SR-3 and SR-4 samples, weight loss decreased at higher temperatures, and this is different from the SR-2 sample, which contained only PbO; thus, there is stability in the weight at higher temperatures.

**Figure 5:**
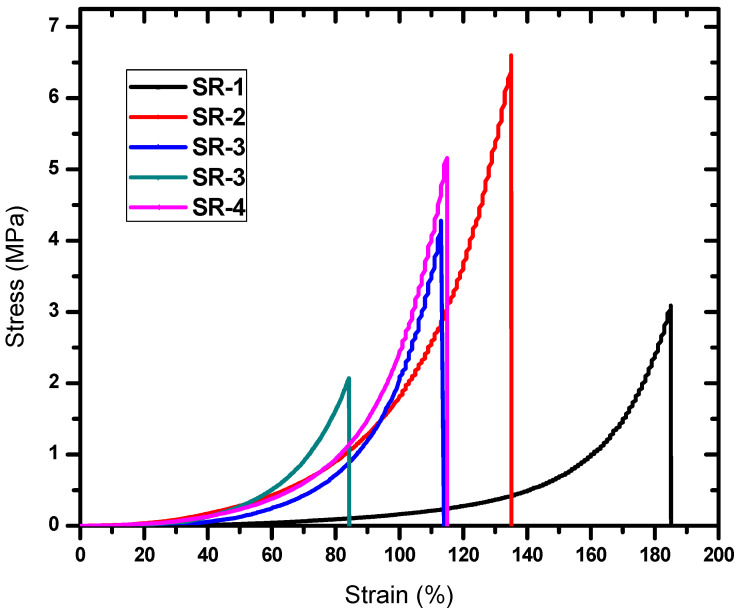
The stress–strain curve of the SR sample in this work.

The stress–strain curve was determined for all five prepared SR materials, as shown in Figure 5. The results showed that the SR-1 sample was more flexible compared to the rest of the other materials, as the elongation rate was 185% at a stress of 3.1 MPa, while the fifth sample (SR-5) had an elongation rate of 115% at a stress of 5.2 MPa. This shows that the addition of HMO nanoparticles reduces the elasticity of natural silicone rubber, but it is still flexible and can be used as an alternative to natural silicone rubber in shielding applications against ionizing radiation.

**Figure 6:**
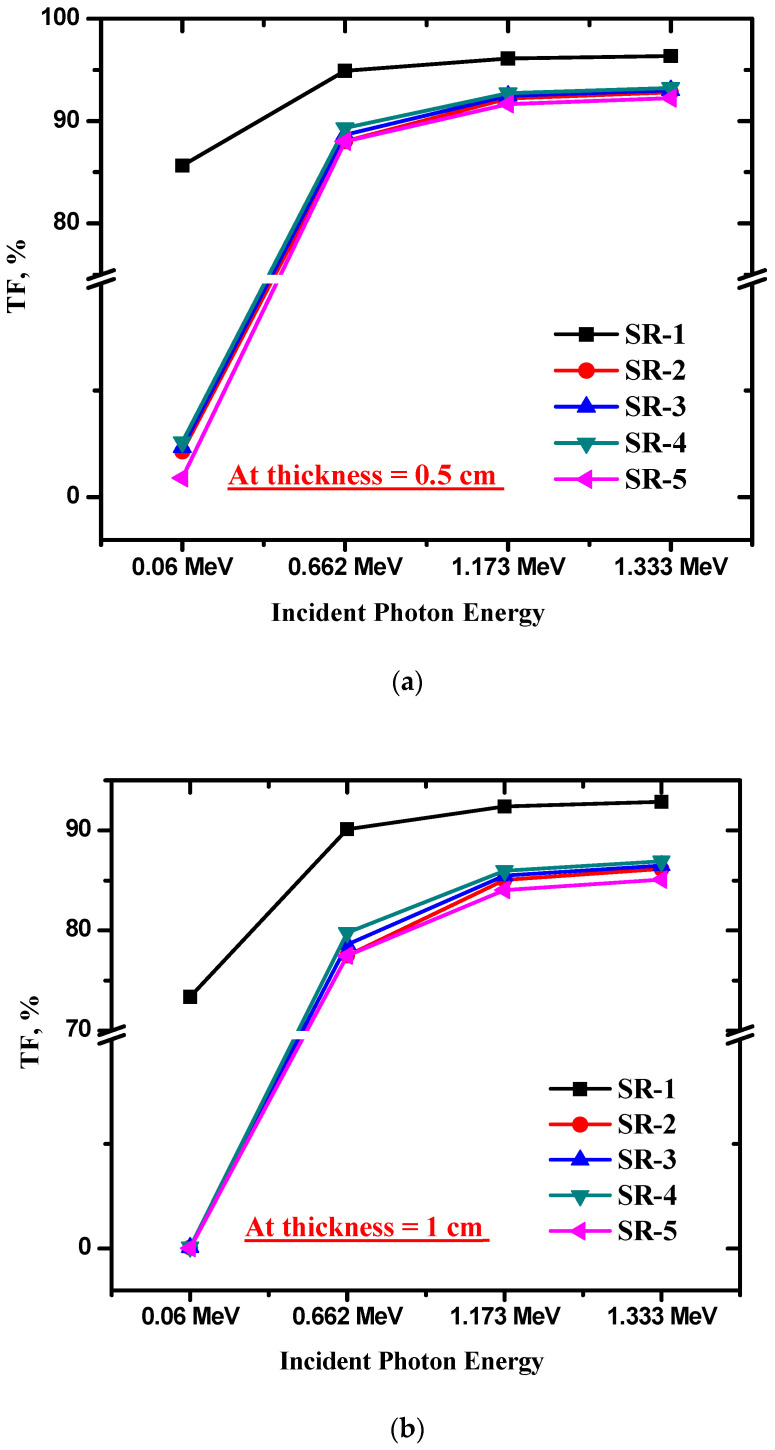

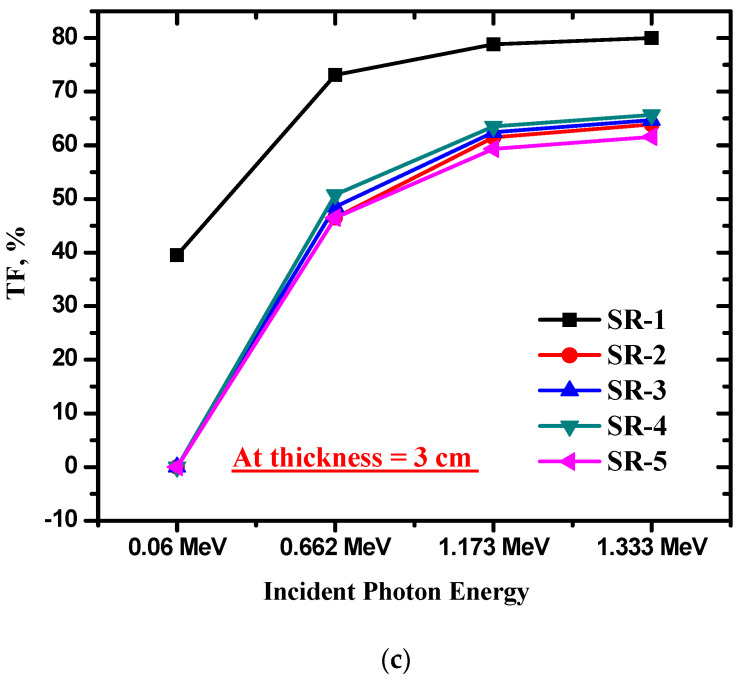
(**a**) Transmission factor at 0.5 cm thickness, (**b**) transmission factor at 1 cm thickness, and (**c**) transmission factor at 3 cm thickness.

For the prepared SR with various dopants, we investigated the TF from the number of photons that reached the NaI detector. This is a basic parameter in the study of radiation shielding since we can directly examine the number of photons that transmit the SR. As well, we can estimate the influence of adding the present oxides (i.e., modifiers) to the number of photons that can transmit the SR. Additionally, from this parameter, we can examine the influence of the thickness of each SR on the TF. So, we can judge the effect of dopants, thickness, energy, and the density of the SR with various oxides on the radiation shielding performance of the prepared SR. We plotted the TF for the SR-1 (pure SR) and the TF for the SR with various dopants in Figure 6a. In this figure, we selected a sample of thickness 0.5 cm from each composition. It is evident that the TF for SR-1 is much higher than that of the remaining SR samples. The TF for SR-1 spans from 85.65 to 96.35%, and this means that most of the photons (even the photons with very low energy) can penetrate the SR sample. At high energy (1.333 MeV, for example), the TF is 96.35%, which means that this sample can only block 3.65% of the photons. For this reason, we tried to introduce some of the heavy metal oxides to the pure SR. It is clear that the inclusion of any of the present HMOs in the SR causes a reduction in the TF value, which means an improvement in the radiation shielding performance. Additionally, the TF depends on the type of HMO used. For instance, the following values are the TF for the prepared samples at 0.06 MeV: 2.13, 2.33, 2.59, and 0.9% for SR-1, SR-2, SR3, SR-4, and SR-5, respectively. These values mean that the SR sample containing 15% of Bi_2_O_3_, WO_3_, BaO, and Zr_2_O_3_ can attenuate almost all the incoming radiation with an energy of 0.06 MeV. Comparing the TF for the SR-1 to SR-5 composites at 0.06 MeV, we found the following order: SR-4 > SR-3 > SR-2 > SR-5. The best attenuator among these composites at 0.06 MeV is SR-5. This is related to the presence of heavy metal oxides in this sample and, at the given low energy (i.e., 0.06 MeV), the photoelectric effect, which has a high dependence on the atomic number and is very important. Accordingly, the presence of Bi, W, Ba, and Zr increases the probability of the occurrence of this process, and this reduces the number of photons that can penetrate SR-5.

We also investigated the role of the thickness of the SR-1 to SR-5 samples on the TF in Figure 6b,c. For SR-1, the TF at 0.06 MeV is 73.37 and 39.49% for a thickness of 1 and 3 cm, respectively. This is evident in the effect of increasing the thickness in enhancing the attenuation ability of the pure SR. The same result is observed for the SR with various HMOs. It is important to mention that the TF at 0.06 MeV is almost 0 for all SR samples with the selected HMOs, which indicates that the SRs with PbO, Bi_2_O_3_, WO_3_, BaO, and Zr_2_O_3_ are able to stop all the radiation with low energy (i.e., 0.06 MeV). When the energy increases to 0.662 MeV, we found that the TF for pure SR-1 was 90.10 and 73.13% for thicknesses of 1 and 3 cm, while, for the SR with the HMOs, we found that the TF for a thickness of 1 cm was about 77%, and in the range of 46.49–50.78% when the thickness was 3 cm. So, when the thickness of these materials is 3 cm, then they can attenuate almost 50% of the incoming radiation with an energy of 0.662 MeV. Therefore, if we need to use these materials to provide enough protection from radiation with an energy of 0.662 MeV, then we must use a sample with a thickness of more than 3 cm. For the Co-60 point source, we found that the TF is about 84% when the thickness used is 1 cm, and about 64% for a thickness of 3 cm. These values are for SR with PbO, Bi_2_O_3_, WO_3_, BaO, and Zr_2_O_3_. Therefore, we must increase the thickness to shield the photons emitted from the Co-60, or we can use two or three layers from a certain composite.

**Figure 7:**
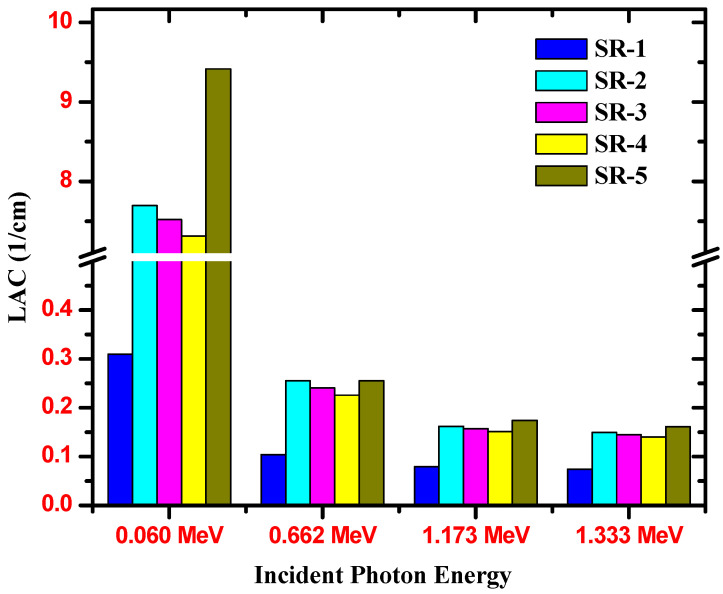
The linear attenuation coefficient for the SR samples and the selected HMOs.

We evaluated the LAC for the pure SR and for the SR with various types of HMOs. The results of the selected energies are presented in Figure 7. The influence of the HMO on the LAC is clear when we compare the LAC of SR-1 (pure SR) with other SRs with the HOM. At 0.06 MeV, the LAC for the SR-1 is 0.3097 cm^−1^, while it sharply increases to 7.696, 7.518, 7.310, and 9.414 cm^−1^ for SR-2 to Sr-5, respectively. This again shows the importance of incorporating the HMO into the prepared samples to increase the probability of photon interactions with these atoms and thus improve the radiation protection effectiveness of the SR samples. Now, we can try to compare the LAC for the SR-2 to SR-5 samples to see the effect of each of the selected HMOs. We can see that the LAC follows this order: SR-5 > SR-2 > SR-3 > SR-4. This is an interesting result, since we found that the sample with the lowest density has the highest LAC, while it is known that the lowest density means the lowest LAC. We can explain this interesting result according to the K-absorption edge of the heavy elements. SR-5 contains Bi, W, Ba, and Zr. The K-absorption edge of these elements is, respectively, 90.5, 69.5, 37.4, and 16 keV.

**Figure 8:**
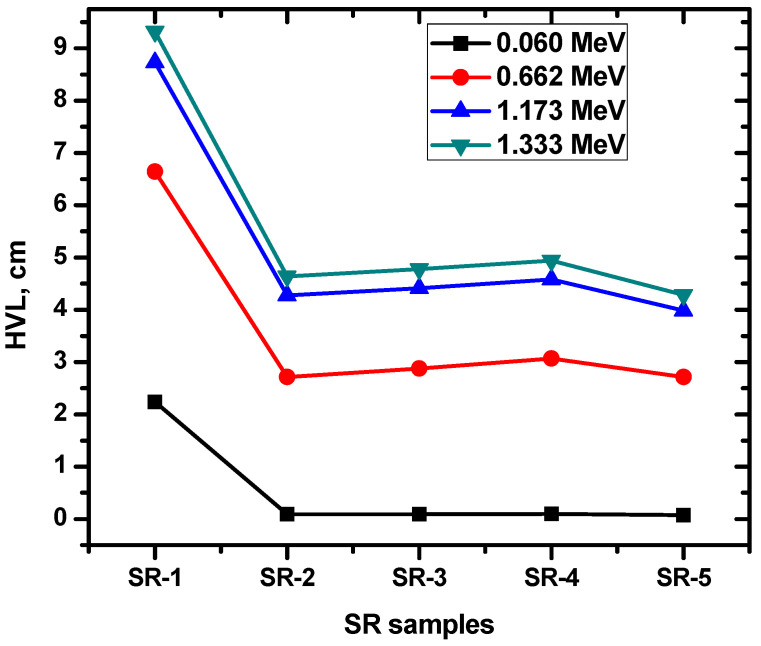
The half-value layer for the SR samples and the selected HMO.

Moreover, the present study aimed to examine the influence of Zr_2_O_3_, BaO, WO_3_, Bi_2_O_3_, and PbO on the half-value layer (HVL) of SR samples. The HVL was evaluated for both pure SR and SR samples containing the aforementioned HMOs at selected energies. The obtained results are illustrated in Figure 8 and demonstrate the impact of these HMOs on the radiation shielding properties of the SR samples. The results showed that the pure SR sample (SR-1) had a higher HVL compared to SR samples containing Zr_2_O_3_, BaO, WO_3_, Bi_2_O_3_, or PbO (SR-2 to SR-5). Specifically, the HVL was lower in the presence of these HMOs, indicating an enhancement in the radiation shielding properties of the SR samples.

At an energy of 0.06 MeV, the HVL for the pure SR sample (SR-1) containing free HMO was found to be 2.238 cm. In contrast, the HVL values for SR-2 to SR-5 were significantly lower, measuring 0.0901, 0.0902, 0.0948, and 0.0736 cm, respectively. These results demonstrate a substantial improvement in the radiation shielding properties of the SR samples when HMOs are incorporated. There is a noticeable discrepancy in the HVL values between the pure SR sample (SR-1) and the other SR samples containing HMOs, indicating a significant effect on the radiation shielding properties of the SR samples. Our results indicate that the addition of HMOs to SR samples leads to a reduction in the HVL at both low and high energy levels, with a more significant effect observed at lower energies. Specifically, the pure SR sample exhibited higher HVL values compared to SR samples containing HMOs, particularly at low energy levels, indicating that the presence of HMO has a greater impact on radiation shielding properties in this range. These observations are likely due to the photoelectric effect, which is known to be more pronounced at lower energies. Comparing the HVL values of the SR samples containing HMO (SR-2 to SR-5), we observed that SR-5, which contains 15% of Bi2O3, WO3, BaO, and Zr2O3, each, had the lowest HVL among the group. In contrast, SR-2, SR-3, and SR-4 had higher HVL values. This difference in HVL can be attributed to the composition of SR-5, which includes multiple HMOs in equal proportions.

**Figure 9:**
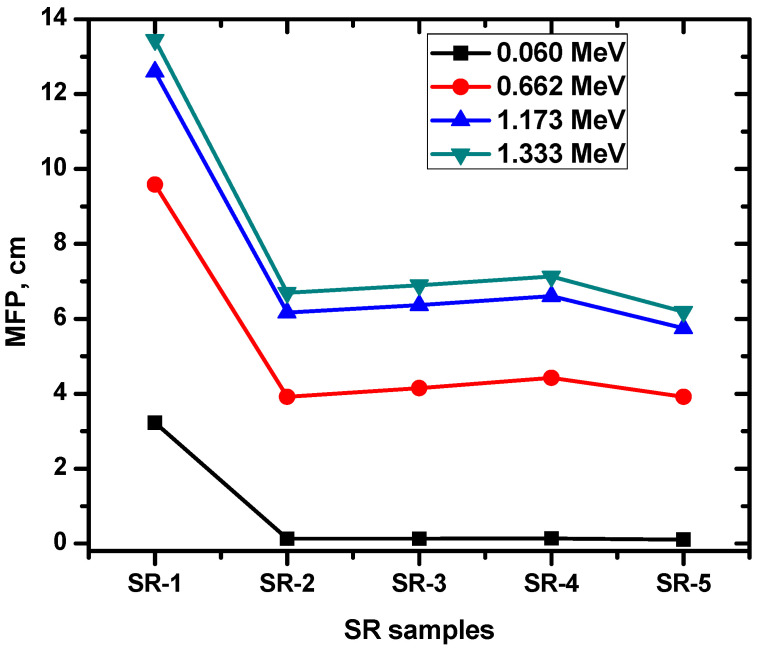
The mean free path for the SR samples and the selected HMO.

In addition to the analysis of HVL, this study investigated the impact of Zr_2_O_3_, BaO, WO_3_, Bi_2_O_3_, and PbO on the mean free path (MFP) of SR samples. The MFP was evaluated for pure SR and SR samples containing the aforementioned HMO at various energy levels. The impact of Zr_2_O_3_, BaO, WO_3_, Bi_2_O_3_, and PbO on the MFP of SR samples was investigated, and the obtained results are presented in Figure 9. The results demonstrate the influence of these HMOs on the radiation shielding properties of the SR samples. The MFP was lower for the SR samples containing Zr_2_O_3_, BaO, WO_3_, Bi_2_O_3_, and PbO (SR-2 to SR-5) compared to the pure SR sample (SR-1). This indicates that the incorporation of these HMOs may contribute to improving the radiation shielding effectiveness of the SR samples. At 0.06 MeV, we evaluated the MFP for pure SR and SR-2 to SR-5 samples. Our findings indicate that the MFP was significantly lower for SR samples containing HMOs compared to the pure SR sample, with values of 0.129, 0.133, 0.137, and 0.106 cm for SR-2 to SR-5, respectively. In contrast, the MFP for the pure SR sample was 3.229 cm. At 0.662 MeV, we also evaluated the MFP for pure SR (SR-1) and SR-2 to SR-5 samples. Our results indicate that the MFP for pure SR was higher, measuring 9.587 cm, compared to the MFP values for SR samples containing HMOs, which were in the range of 3.91 cm to 4.43 cm. At 1.173 MeV, the MFP for pure SR (SR-1) was almost double the MFP values for SR samples SR-2 to SR-5. Specifically, the MFP for SR-1 was 12.59 cm, while the MFP values for SR-2 to SR-5 ranged from 5.74 cm to 6.60 cm. These results again highlight the significant effect of HMO on the MFP and radiation shielding properties of the SR samples. Our MFP results suggest that developing radiation shielding materials incorporating multiple HMOs may be beneficial. Specifically, we observed that SR-5, which contains 15% of Zr_2_O_3_, BaO, WO_3_, and Bi_2_O_3_, had a lower MFP than the other SR samples. These findings support the notion that the combination of different HMOs may lead to improved radiation shielding properties.

**Figure 10:**
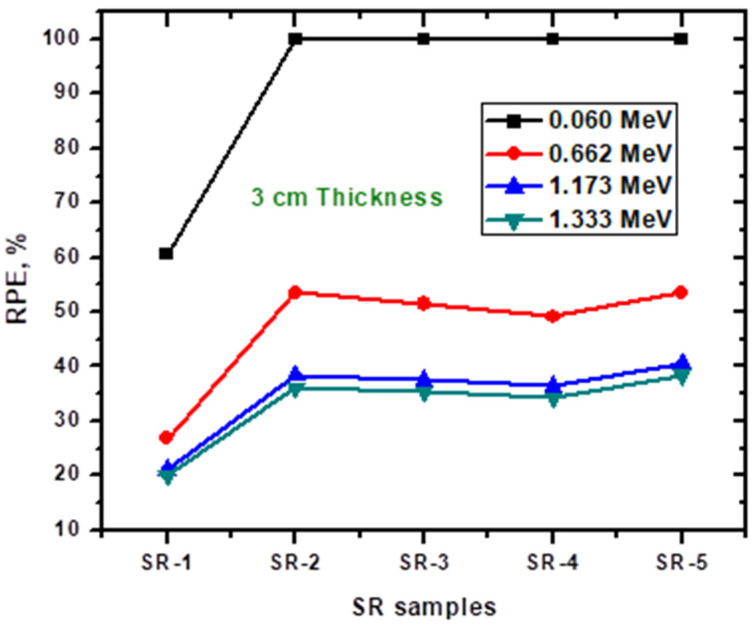
The radiation protection efficiency for the SR samples and the selected HMO.

In order to comprehensively evaluate the radiation shielding performance of the SR samples, we calculated the RPE for a thickness of 3 cm and visualized the results in a plot (Figure 10). This analysis provides a clear understanding of the shielding effectiveness of each sample, facilitating a direct comparison of the radiation attenuation properties of each sample.

At 0.06 MeV, the RPE for the pure SR sample (SR-1) was found to be 60%, indicating that 40% of the incoming radiation-carrying energy of 0.06 MeV can penetrate the SR-1 sample. Therefore, SR-1 can block only 60% of the intensity of the radiation at this energy level. In contrast, the SR samples containing HMO (SR-2 to SR-5) demonstrated an RPE of 100%, suggesting that these samples are capable of attenuating all radiation-carrying energy of 0.06 MeV. The observed increase in radiation shielding effectiveness with the incorporation of HMO highlights the potential advantages of using these additives in developing more efficient radiation shielding materials. At 0.662 MeV, the RPE for the pure SR sample (SR-1) significantly decreased to 26.87%. In contrast, the RPE for the SR samples containing HMO (SR-2 to SR-5) also decreased, but to a lesser extent. Specifically, the RPE was found to be 53.5%, 51.4%, 49.2%, and 53.5% for SR-2, SR-3, SR-4, and SR-5, respectively. These results suggest that the addition of HMO in the SR samples can lead to a significant improvement in their radiation shielding properties, particularly at lower energies. As evidenced by the previous results, the RPE for the pure SR sample (SR-1) was found to be lower than that of the SR samples containing HMO. Among the HMO-containing samples (SR-2 to SR-5), SR-5 demonstrated a slightly higher RPE compared to SR-2, SR-3, and SR-4. This could be attributed to the specific chemical composition of SR-5, which contained Bi, Ba, W, and Zr. The high atomic numbers of these elements increase the likelihood of photon interactions, leading to greater attenuation of the radiation. In general, these data underscore how important it is to maximize the efficacy of radiation shielding materials by adjusting the chemical composition of the shielding material.

## 4. Conclusions

In conclusion, this study investigated the potential of heavy metal oxide (HMO) nanoparticles as modifiers to improve the radiation shielding properties of silicone rubber (SR) composites. The prepared SR composites were characterized for their thermal, mechanical, and radiation shielding properties and the results demonstrated that the addition of HMO nanoparticles enhanced the thermal stability and radiation shielding performance of the composites. Specifically, the TF decreased with the addition of HMO nanoparticles, indicating improved attenuation effectiveness. The HVL and MFP were also significantly reduced with the addition of HMO, indicating enhanced radiation shielding properties. SR-5, which contained a combination of Zr_2_O_3_, BaO, WO_3_, and Bi_2_O_3_, showed the best radiation shielding properties among the prepared composites. Our findings suggest that developing radiation shielding materials incorporating multiple HMOs may be beneficial for enhancing their radiation shielding properties. Furthermore, the density, ultimate deformation displacement, and thermal stability of the prepared composites were also characterized, providing insight into their mechanical properties. The density of the composites ranged from 1.25 to 2.611 g·cm^−3^, with SR-2 having the highest density due to the presence of lead oxide. The ultimate deformation displacement ranged from 14.17 to 21.23 mm, with the highest value recorded for SR-3 and the lowest for SR-2. Additionally, the thermal stability of the materials improved with the addition of HMO nanoparticles. Overall, this study provides valuable insight into the development of silicone rubber composites with heavy metal oxide nanoparticles for gamma ray shielding applications. The prepared composites exhibited promising radiation shielding properties, indicating their potential use in various radiation shielding applications. Further research could investigate the optimization of the composition and structure of the composites to further enhance their radiation shielding effectiveness.

## Figures and Tables

**Table 1:** Properties of presented HMOs-NPs.

Property	Bi_2_O_3_-NPs	WO_3_-NPs	BaO-NPs	Zr_2_O_3_-NPs
Appearance (Color)	White to Yellow	Yellow	White	White
Appearance (Form)	Powder	Powder	Powder	Powder
Avg. Size (TEM)	20 ± 5 nm	35 ± 5 nm	20 ± 5 nm	21 ± 5 nm
Shape (TEM)	Spherical Shapes	Spherical Shapes	Spherical Shapes	Mixture of Spherical and Quasi-Spherical Shapes

**Table 2:** The composition and densities of the prepared mixtures.

Code	Composition (wt %)						Density (g·cm^−3^)
	Silicone Rubber (SR)	PbO	Bi_2_O_3_ NPs	WO_3_ NPs	BaO NPs	Zr_2_O_3_ NPs	
SR-1	100	—	—	—	—	—	1.250
SR-2	40	60	—	—	—	—	2.611
SR-3	40	40	5	5	5	5	2.555
SR-4	40	20	10	10	10	10	2.500
SR-5	40	—	15	15	15	15	2.448

## Data Availability

All data generated or analyzed during this study are included in this published article.
